# Water permeability is a measure of severity in acute appendicitis

**DOI:** 10.1080/14756366.2017.1347167

**Published:** 2017-08-01

**Authors:** Nicola Pini, Viktoria A. Pfeifle, Urs Kym, Simone Keck, Virginie Galati, Stefan Holland-Cunz, Stephanie J. Gros

**Affiliations:** Department of Pediatric Surgery, University Childrens’ Hospital of Basel (UKBB), Basel, Switzerland

**Keywords:** Aquaporin 1, water permeability, enteric nervous system, appendicitis, pediatric surgery

## Abstract

Acute appendicitis is the most common indication for pediatric abdominal emergency surgery. Determination of the severity of appendicitis on clinical grounds is challenging. Complicated appendicitis presenting with perforation, abscess or diffuse peritonitis is not uncommon. The question remains why and when acute appendicitis progresses to perforation. The aim of this study was to assess the impact of water permeability on the severity of appendicitis. We show that AQP1 expression and water permeability in appendicitis correlate with the stage of inflammation and systemic infection parameters, leading eventually to perforation of the appendix. AQP1 is also expressed within the ganglia of the enteric nervous system and ganglia count increases with inflammation. Severity of appendicitis can be correlated with water permeability measured by AQP1 protein expression and increase of ganglia count in a progressive manner. This introduces the question if regulation of water permeability can present novel curative or ameliorating therapeutic options.

## Introduction

Acute appendicitis is the most common indication for emergency abdominal surgery in children^1^^,^^2^. Advances in the technology and improved access to imaging modalities have changed the diagnostics and management of acute appendicitis^3^^,^^4^. The clinical evaluation of the severity of appendicitis represents a challenge for the surgeon and can determine the treatment^5^. Complicated appendicitis presenting with perforation, abscess, or diffuse peritonitis is not uncommon and is reported in up to 30–50% in some studies^6^^,^^7^. The risk of fast progression of the disease and perforation is especially high in young children^7^. Albeit several studies on clinical parameters as well as prognostic serum markers candidates e.g. infection markers, fibrinogen etc.^8^, the question still remains why and when appendicitis leads to perforation.

We hypothesize that water permeability along with one of its key players namely the water channel protein AQP1, the first molecular water channel described^9^), might play an important role in the process of enteric wall perforation in progressive appendicitis. We hypothesize that an increase in water permeability together with an increasing inflammatory process leads to a circle of increased enteric wall permeability, transgression of bacteria, enteric wall edema, hypoxia and eventually perforation.

Aquaporins are a family of water channels that are responsible for water transport through membranes and are expressed in various epithelial and endothelial tissues as well as in the peripheral and central nervous system^10^. The different AQP isotypes take part in several functional processes such as phagocytosis, apoptosis, migration and neuronal signal transduction^10^^,^^11^. While the function of several AQP channels in the central nervous system is actively researched, their expression and function in the digestive tract and especially the enteric nervous system is still largely unknown. A connection between AQP1 expression, as a measure of water permeability, and acute inflammation of the appendix has not been examined before.

## Materials and methods

### Patients

Forty tissue samples from patients with the diagnosis of appendicitis that underwent appendectomy at the University Children’s Hospital Basel between March 2016 and July 2016 were used for this study. Written consent of the patient’s legal guardian was obtained prior to the operation. The use of human tissue was in accordance with the ethics approval by the Ethics Commission North-West-Central Switzerland in Basel (EKNZ 2015-263). The stage of inflammation was assessed by routine scoring at the time of surgery by the surgeon as well as by routine H.E. staining-based pathological evaluation and categorized as acute, phlegmonous, or perforated.

### Tissue preparation

The appendix specimens for this study were prepared according to a modification of the “Swiss role” as described by Meier-Ruge and Bruder for histomorphological examination of the intestine^12^. This “appendix role” technique allows the examination of a longitudinal section from the base to the tip of the appendix on one slide. Appendices were cut longitudinally and rolled into a snail with the serosa facing in and the mucosa facing out of the role. The tissue was embedded in OCT (TissueTek) and snap frozen.

### Immunohistochemical and immunofluorescence staining

**Figure 1.**
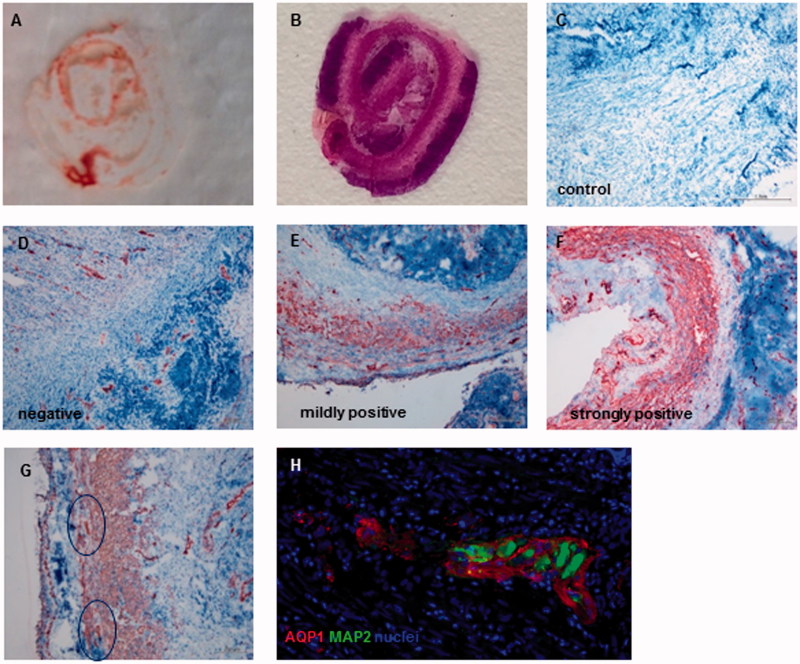
(A) Cryosection and (B) H.E. staining of appendix role. (C–F) Representative images of control, AQP1 negative, AQP1 mildly positive and strongly positive immunohistochemical staining (10× standard microscopic enlargement) (G) Consecutive pictures of tip and base were taken (10× standard microscopic enlargement) and AQP1 positive ganglia were counted (H) Immunofluorescence co-staining revealed different expression patterns for AQP1 and MAP2 (40x standard microscopic enlargement).

The samples were cut to a thickness of 7 μm were cut on a cryotome and mounted onto microscope slides displaying the entire appendix role from base to tip (Figure 1(A,B)). For the immunohistochemical AQP1 staining, a HRP-AEC kit (R&D Systems, Minneapolis, MN) was used according to the manufacturer’s protocol using the primary polyclonal rabbit anti-AQP1 antibody (Merck Millipore, Germany) at a dilution of 1:400. The slides were counterstained with a hematoxylin solution (Spitalpharmazie USB Basel, Switzerland). Negative controls using the antibody diluent (DAKO, Denmark) and missing the primary antibody were included in every staining cycle. Slides were analyzed by using an Olympus BX43 microscope. The staining was scored by two independent examiners who were blinded regarding clinical data.

For AQP1 and MAP2 immunofluorescence double staining, the polyclonal rabbit anti-AQP1 antibody (Merck Millipore, Germany) and the monoclonal mouse MAP2 antibody (Abcam, UK) both at a dilution of 1:200 were used following a standardized protocol. As secondary antibodies goat anti-mouse IgG1 AlexaFluor 488 and goat anti-rabbit AlexaFluor 647 were used. Negative controls missing the primary antibody were included in every staining cycle. Slides were mounted using ProLong^®^ Gold Antifade Mountant with DAPI (Life Technologies, Thermo Fisher Scientific Inc., USA). Slides were analyzed with an Olympus BX63 microscope using CellSens software. For the analysis, three consecutive pictures of each base and tip of the resected specimens were recorded at a magnification of 10×, and the ganglia of the enteric nervous system were counted by two independent examiners.

### Statistical analysis

Data were analyzed using the SPSS version 23.0 (SPSS, Chicago, IL), and a *p* values of less than 0.05 was defined as significant. For analyzing the correlation of the diagnosis with AQP1 expression and infection parameters, the Spearman’s-rank-correlation-coefficient was used.

## Results

We examined samples of 40 patients, including 21 males and 19 females, with a mean age of 10 years old ranging from 2.9 to 17.7 years (Table 1). Neither sex nor age had a statistically significant impact on perforation. The laboratory parameters C-reactive protein (CRP) and leucocyte count were available for all patients.

**Table 1.** Patient characteristic.

Patient characteristics		All patients	Acute appendicitis	Phlegmonous appendicitis	Perforated appendicitis	Control
Age at operation	years (mean)	2.9–17.7 (10)	5.5–17.0 (11)	2.9–17.7 (11.3)	8.0–13.0 (9.8)	10.0–16.0 (13)
Sex	f/m (%)	50/50	42.9/57.1	52.6/47.4	40/60	100/0
AQP1 expression	negative	14	8	4	0	2
	mildly positive	14	4	9	1	0
	strongly positive	12	2	6	4	0
Inflammation parameters (CRP/Leucocytes)	none elevated	9	7	0	1	2
	one elevated	19	6	12	1	0
	both elevated	12	1	7	3	0
	total	40	14	19	5	2

a Age and sex ns., correlation of AQP1 expression and inflammation depicted in Figure 2.

### AQP1 expression in inflamed appendices

Overall, AQP1 expression was present in the mucosa, in the longitudinal and circular muscle layers, in the serosa, and in blood vessels in the meso-appendix, which served as an internal positive control. Lymph follicles within the intestinal wall were negative for AQP1 and thus served as an internal negative control. Changes in the AQP1 expression, however, were most prominent in the longitudinal and circular muscle layers. A score combining the intensity and area of AQP1 staining of mucosa, musculature, and serosa was used for assessment. AQP1 expression was categorized as AQP1 negative, mildly positive, or strongly positive (Figure 1). Out of the 40 samples, a total of 14 (35%) were negative, 14 (35%) were mildly positive, and 12 (30%) were strongly positive (Table 1, Figure 1(C–F)). According to their clinical and pathological scoring, they were classified as acute *n* = 14 (35%), as phlegmonous *n* = 19 (47.5%), and as perforated *n* = 5 (12.5%). A significantly positive correlation between the AQP1 expression and the stage of the disease was found (correlation coefficient 0.542, *p* ≤ 0.001) (Figure 2). Furthermore, we defined an inflammation score depending on whether the standard clinical inflammation parameters CRP and/or leucocyte count were elevated. In 9 of these 40 patients (22.5%), neither CRP nor leucocyte count were elevated, in 19 (47.5%) one of the values was elevated and in 12 (30%) both were elevated. We found a significantly positive correlation between these inflammation parameters and the stage of the disease (correlation coefficient 0.575, *p* ≤ 0.001) (Figure 2). Moreover, there was a significant positive correlation between the laboratory inflammation parameter and the expression of AQP1 in the tissue (correlation coefficient 0.347, *p* = 0.028) (Figure 2). Taking these finding together, we show that AQP1 expression and with it water permeability seems to be up-regulated with increasing inflammation locally in the appendix. This local inflammation correlates with systemic infectious parameters in the blood.

**Figure 2.**
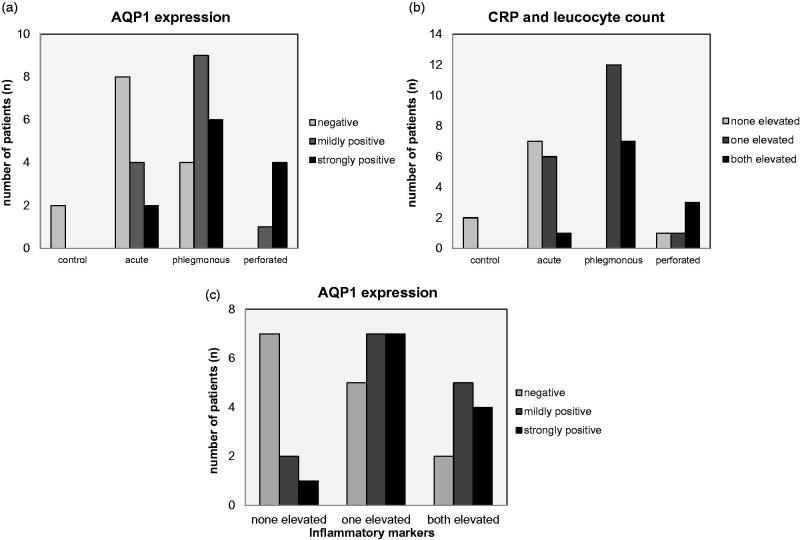
Correlation of the stage of the disease with AQP1 expression and CRP and leucocyte count A significantly positive correlation of the stage of the disease with the AQP1 expression (correlation coefficient 0.542, *p* ≤ 0.001) (A) as well as with CRP and Leucocyte values (correlation coefficient 0.575, *p* ≤ 0.001) (B) is shown. Moreover, a significantly positive correlation of laboratory inflammation parameters with AQP1 expression in the tissue (correlation coefficient 0.347, *p* = 0.028) (C) could be found.

### Enteric nervous system ganglia of the appendix and AQP1

Furthermore, prominent changes in the enteric nervous system were observed in the inflamed specimen regarding number and localization of ganglia. 23 inflamed appendices were further examined. Ganglia count showed that 65.2% of the specimens had a higher ganglia count in the inflamed region, namely the tip of the appendix, compared to the not inflamed region (Figure 1(G), Table 2). When combining staining for AQP1 with staining for the neuronal ganglion marker MAP2^13^, we could show that although AQP1 is located within the ganglia it is not co-localized with MAP2. (Figure 1(H)). Our findings show that AQP1 is located in ganglionic fibers surrounding MAP2-positive neurons of the enteric nervous system of the appendix.

**Table 2.** Distribution of ganglia.

Ganglion count	Specimen with higher total number of ganglia (*n*)	Percent (%)
Base	4	17.4
Tip	15	65.2^a^
No difference	4	17.4
Total	23	100

a Significantly positive correlation of higher ganglion count with location in tip (inflamed region).

## Discussion

In this study, we found a positive correlation of AQP1 expression in progressive appendicitis, and with this water permeability of the entire enteric wall, with the stage of inflammation. The increase of water permeability appears to eventually be associated with perforation of the appendix wall. Furthermore, AQP1 is expressed within the ganglia of the enteric nervous system of the appendix and inflammation appears to be associated with an increase of ganglia within the inflamed region. Moreover, a significant positive and continuous correlation between CRP and leucocyte count and AQP1 expression was found.

### Water permeability and inflammation

The expression of the water channel AQP1 has been shown to be essential in many different physiological processes throughout the whole body. The major function of APQ1 is maintaining and regulating water homeostasis. There is previous evidence that APQ1 plays a role in some aspects of the peripheral nervous system^10^^,^^14^. We show here that in the progression of appendicitis there is an increase of AQP1 expression and, inevitably associated with this, an increase of water permeability of the entire enteric wall in the inflamed region. We can only speculate whether the inflammation of the appendix leads to an increase of APQ1, or a primary up-regulation of AQP1 in the appendix leads to an increase of intestinal wall permeability and susceptibility to bacteria transgression and organ infection. The clinically relevant outcome in both cases is an increase in water influx into all layers of the appendix wall, leading to edematous transformation, probably hypoxia and perforation.

In tumor disease, for example, there is evidence that up-regulation of AQP1 expression is linked to hypoxia^15–17^. In accordance with prior findings of Rodríguez-Sanjuán et al., we found a significant positive and continuous correlation between CRP and leucocyte count and the stage of the inflammation^18^. Nonetheless, laboratory infection parameters individually remain weak diagnostic markers and cannot replace but only supplement the thorough clinical examination of the typical clinical presentation by an experienced surgeon^19^. We, however, also found a significant positive correlation of systemic inflammation values and the expression of AQP1 in the organ. This strengthens a theory of a progressive course of the disease. One might even hypothesize that by selective inhibition of AQP1 and water permeability, perforation could be kept at bay.

### Expression of AQP1 in the enteric nervous system

While the function of several AQP isotypes in the central nervous system is being actively researched, their expression and physiological function in the digestive tract and especially in the enteric nervous system is still largely unknown and results are controversial. Gao et al. first reported a strong AQP1 protein expression in the submucosal and myenteric nerve plexus in the human esophagus. AQP1 is co-localized with S-100, indicating glial cell-specific AQP1 expression, while neurons in the ganglia were not positive for AQP1^20^. The neuronal expression pattern of AQP1 in rat ileum however was reported to present strong expression of AQP1 in a subset of neurons in both myenteric and submucosal plexus of the enteric nervous system^21^. In this study, 9% of neurons that were positive for the neuronal marker HuC/D expressed AQP1. The localization of AQP1 in neurons and fibers of rat myenteric nerve plexus was confirmed by a further study^22^. Contrary to this, Arciszewski et al. confirmed the neuronal expression pattern observed in the rat ileum, but found AQP1 in the ovine duodenum to be mainly expressed in the submucosal but not in the myenteric plexus^23^. This last study further hypothesizes that some AQP1 immuno-reactive neurons may function as sensory neurons.

The distribution of the AQP1 water channels in the enteric nervous system could suggest a functional involvement of AQP1 in regulating water homeostasis, intestinal motility, mucosal transport and enteric pain perception^20^. Rare AQP1-deficient humans did not present with overt gastrointestinal symptoms^24^. However, the distribution pattern of AQP1 immuno-reactivity in the enteric nervous system appears to be species-specific. The expression of AQP1 has not been conclusively addressed in the entire intestine of any species.

In our pediatric patient appendices we found AQP1 positive ganglia most prominently but not exclusively in the myenteric plexus. Within the ganglia, AQP1-positive cells did not co-localize with MAP2-positive neurons, which is in agreement with earlier findings by Gao et al.^20^. Although the functional aspect of AQP1-mediated water homeostasis has not been addressed yet, AQP1 may well be involved in the physiological and pathophysiological neuronal activity by regulating water homeostasis in the ganglia and nerve fiber bundles of the enteric nervous system.

A further functional aspect of nerve function evolves around pain perception. AQP1 has been found to be an important factor in axonal growth and the regeneration of dorsal root ganglion neurons which transduce peripheral pain signals through small-diameter, non-myelinated C-fibers^17^. Our histopathological finding of an increased number of ganglia in the inflamed region of the appendix could strengthen a role of AQP1 positive ganglia in the process of pain signaling and lead to interesting future investigations. It presents a challenge to utilize targeted inhibition of AQP1 in the treatment of this disease.

### Targeting AQP1

There are several inhibitors of AQP1 that are currently being evaluated. Amongst the quaternary ammonium based compounds TEA is the lead component in blocking AQP1. It is more selective for AQP1 than for potassium channels and is highly effective regarding water permeation^25^. TEA potently and reversibly inhibits water permeation without influencing AQP1 plasma membrane expression levels^25^^,^^26^. The bumetanide derivatives AqB007 and AqB011 represent selective blockers of AQP1 ion channel conductance^27^, and a few further chemical compounds have been tested as AQP1 inhibitors^28^. Although none of these inhibitors has entered clinical testing as AQP1 inhibitor yet, efforts are being made towards development of possible therapeutics.

## Conclusions

Severity of appendicitis and of systemic inflammation parameters can be positively correlated with water permeability, as measured by AQP1 protein expression in a progressive manner. Inflammation correlates with an increased ganglia count in the enteric nervous system, in which AQP1 is present in MAP2-negative cells. Both, increase in AQP1 expression and in ganglia count, strengthen the theory of a progressive course of the disease. This might in the future lead to novel curative or ameliorating treatment options.
